# Prognostic Value of MiR-21: An Updated Meta-Analysis in Head and Neck Squamous Cell Carcinoma (HNSCC)

**DOI:** 10.3390/jcm8122041

**Published:** 2019-11-21

**Authors:** Alexandra Iulia Irimie-Aghiorghiesei, Cecilia Pop-Bica, Sebastian Pintea, Cornelia Braicu, Roxana Cojocneanu, Alina-Andreea Zimța, Diana Gulei, Ondřej Slabý, Ioana Berindan-Neagoe

**Affiliations:** 1 Department of Prosthetic dentistry and Dental materials, Division Dental Propaedeutics, Aesthetic, “Iuliu Hatieganu” University of Medicine and Pharmacy, 400337 Cluj-Napoca, Romania; irimie.alexandra@umfcluj.ro; 2 Research Center for Functional Genomics and Translational Medicine, “Iuliu Hatieganu” University of Medicine and Pharmacy, 400337 Cluj-Napoca, Romania; cecilia.bica8@gmail.com (C.P.-B.); braicucornelia@yahoo.com (C.B.); cojocneanur@gmail.com (R.C.); 3 Department of Psychology, Babes-Bolyai University, 400015 Cluj-Napoca, Romania; sebastianpintea@psychology.ro; 4 MEDFUTURE-Research Center for Advanced Medicine, “Iuliu Hatieganu” University of Medicine and Pharmacy, 400337 Cluj-Napoca, Romania; andreea.zimta@umfcluj.ro (A.-A.Z.);; 5 Centre for Molecular Medicine, Central European Institute of Technology, Masaryk University, 62500 Brno, Czech Republic; on.slaby@gmail.com; 6 Department of Comprehensive Cancer Care, Masaryk Memorial Cancer Institute, Faculty of Medicine, Masaryk University, 62500 Brno, Czech Republic; 7 Department of Functional Genomics and Experimental Pathology, The Oncology Institute “Prof. Dr. Ion Chiricuta”, 400015 Cluj-Napoca, Romania

**Keywords:** miR-21, head and neck cancer, survival, prognostic, bioinformatics analysis

## Abstract

Head and neck squamous cell carcinoma (HNSCC) is a group of malignancies with serious impact on patient quality of life due to a reduced rate of response to chemotherapy or radiation therapy. MiR-21 has been identified as one of the most common proto-oncogenes. It is hypothesized that upregulated miR-21 could serve as a potential biomarker for human cancer diagnosis. Considering the target genes identified for miR-21 in HNSCC, this transcript is an important player in several cellular processes that control carcinogenesis. The abnormal expression of miR-21 in this group of pathologies has been assessed in several publications, but given the heterogeneity of the published results, a meta-analysis and proper bioinformatics analysis of expression databases are needed to correctly establish the prognostic potential of this molecule. The present meta-analysis comprises the published survival data on HNSCC patients, reported as HR and 95% CI, in association with the expression levels of miR-21. Our investigation revealed that miR-21 could be used successfully as a prognostic biomarker in HNSCC patients, confirming its oncogenic potential. Specifically, the upregulation of miR-21 in these patients predicts a worse outcome in terms of survival rate.

## 1. Introduction

Head and neck cancer (HNC) is considered a pathology with severe consequences on the quality of life of patients, with a reduced rate of response to treatment [1,2]. HNC is a highly heterogeneous group of tumors occurring on the mucosal surfaces of the nasal and oral cavity, oropharynx, and larynx [1]. From this heterogeneous group, over 90% of diagnosed cases are head and neck squamous cell carcinomas (HNSCCs), statistically being the sixth most widespread cancer worldwide [1].

The survival rate remains low, since up to 25% of the patients progress to metastatic disease. The presence of the lymph node metastases (LNM) represents an important determinant factor in HNSCC, considering the positive correlation between the lymphatic metastatic spread and the survival rate [3]. The main risk factors are related to smoking and alcohol consumption [4,5]. Also, infection with high-risk human papillomaviruses (HPVs) has recently been associated with the pathogenesis of HNSCC [6].

Considering the complexity of the HNSCC molecular mechanism, most studies have focused on coding and noncoding genes. MicroRNAs (miRNAs) are noncoding transcripts, 18–22 nucleotide long RNAs, being vital posttranscriptional regulators of gene expression [7]; at the cellular level they might act as oncogenes or tumor suppressor genes affecting cell proliferation, invasion, migration, self-renewal capacity, resistance to therapy, and autophagy [8,9].

This noncoding RNA is upregulated in various human cancers [10,11], including HNSCC [12]. MiR-21 regulates the expression of multiple target genes with fundamental implications in cancer progression [13]. In oral squamous cell carcinoma, this microRNA is part of the mechanism that regulates sensitivity to cisplatin, by targeting phosphatase and tensin homolog (PTEN), as well as programmed cell death protein 4 (PDCD4) genes. Moreover, this feature is transmissible through the presence of exosomes carrying miR-21 to other cells [14], hence supporting previous findings [15]. Comparable results were reported by Yan et al., 2018, in salivary adenoid cystic cancer cell lines, with miR-21 being involved in tumor cell growth via the regulation of PDCD4 expression and B-cell lymphoma 2 (Bcl-2) genes. In addition, miR-21 has been shown to target the PTEN gene, being involved also in the ability of salivary adenoid cystic carcinoma cells to metastasize [16]. Another study observed that the overexpression of miR-21 and cyclin-dependent kinase 5 (CDK5) were associated with epithelial-to-mesenchymal transition (EMT) and lymph node metastasis (LNM). The results suggested that miR-21/CDK5 interaction impacted EMT by targeting signal transducers and activators of transcription 3 (STAT3) in HNSCC cells [12]. In tongue squamous cell carcinoma, upregulation of mir-21 could independently predict shorter survival of patients by modulating the expression of the tropomyosin 1 (TPM1) gene and inhibiting the apoptosis of tumor cells [17]. Furthermore, there is evidence regarding the modulation of radiosensitivity through miR-21; it was observed that the downregulation of miR-21 increased the radiosensitivity of nasopharyngeal cell lines [18]. All these investigations emphasize the role of miR-21 in the progression of head and neck pathologies, but also in resistance to treatment, given that radiotherapy is used as the primary treatment along with surgery, as it is confined to local effects [19].

Several studies have focused on evaluating the expression levels of this transcript; however, inconsistencies or heterogeneities concerning the prognostic value of miR-21 remain. Our work focused on performing a meta-analysis in order to assess the expression levels of miR-21 and to accurately evaluate its prognostic value in HNSCC. We also did a statistical analysis of various databases to gain a more in depth understanding of the miR-21 expression across various experiments and of the significance of its inhibitory activity.

## 2. Methods

### 2.1. Search Strategy and Eligibility Criteria for Meta-Analysis

Following the guidelines and criteria of Preferred Reporting Items for Systematic Reviews and Meta-Analyses (PRISMA), we did a systematic search directed by interrogating the PubMed and Embase databases. Our search approach assumed the following terms: (‘otolaryngolog’ OR ‘head and neck’ OR ‘buccal’ OR ‘mouth’ OR ‘oral cavity’ OR ‘larynx’ OR ‘lip’ OR ‘hypopharynx’ OR ‘oropharynx’ OR ‘tongue’) AND ‘patients’ AND ‘survival’ AND (‘miR-21’ OR ‘miRNA 21’ OR ‘microRNA 21’). This strategy was applied for both databases. Studies that investigated the prognostic value of miR-21 expression in the tissue/serum/plasma of HNSCC patients and that were published before May 2019 were assessed for eligibility. We chose only publications that reported hazard ratios (HR) and confidence intervals (CI) for the detailed investigation.

We excluded articles that met any of the following criteria: (1) conference/meeting abstracts; (2) not published in English; (3) full text not available; (4) written as review/meta-analysis; (5) the expression of miR-21 was not evaluated in human samples; (6) did not offer sufficient data regarding the HR and 95% CI for the survival analysis.

### 2.2. Search Strategy in Open Databases

We searched the ArrayExpress database with the terms: “miR-21, head and neck cancer” or “miRNA head and neck cancer”. The study selection was done accordingly: (1) inclusion of human subjects; (2) expression analysis done on normal and tumor tissue; (3) the localization of tumors must include the head and neck area; (4) the expression analysis should include miR-21 expression analysis; (5) exclusion of unprocessed datasets; (6) datasets with at least 10 samples. Addition datasets were included based on a similar study done on miR-375-5p in head and neck cancer [20]. We also searched the TCGA database and retrieved the miRNA Expression Quantification (*n* = 569) GDC Hub.

In order to assess the expression level of these genes, especially the genes targeted by miR-21, we retrieved data from the GDC and TCGA Head and Neck Cancer (HNSC) datasets, which included HTSeq-Counts (*n* = 546).

### 2.3. Quality Appraisal

The eligibility criteria were separately employed by two individual reviewers who then proceeded to data extraction (C.P.-B. and R.C.). Studies were selected for analysis if they met the following benchmarks: (1) the expression of miR-21 was determined in patients with HNSCC; (2) the assay used to quantify the expression was clearly defined; (3) the outcome of patients was expressed as overall survival (OS), relapse-free survival (RFS), cancer-specific survival (CSS), disease-free survival (DFS) or 5-year survival; (4) the HRs of the obtained miR-21 are clearly explained as high vs. low/low vs. high.

### 2.4. Data Extraction

The information retrieved from the selected articles was organized in an excel worksheet that included data about publication (DOI, author’s name), population (country, smoking status, age), sample type and count, tumor data (anatomical site, staging, lymph node invasion, human papillomavirus (HPV) status, experiment (assay, miRNA expression), and statistical analysis (HRs, 95% CI, *p*-value, survival type, direction of result).

### 2.5. Data Analysis 

Analyses were conducted by using Comprehensive Meta-Analysis software, version 2.2.050 (Biostat Inc., Englewood, NJ, USA). As an indicator of effect sizes, the hazard ratio was used. Given the nonsignificant heterogeneity of the studies, all analyses were based on a fixed effects model.

Publication bias analysis was performed using two methods. First, we used the Classic Fail-safe N of Rosenthal, in order to evaluate the number of studies with null effects that are required to turn the effect size to zero. Second, we calculated the Begg and Mazumdar’s rank correlation test. This test computes the rank order correlation (Kendall’s tau b) between the effect size and the standard error (which is driven primarily by sample size). This determines whether large studies tend to be included in the analysis, regardless of their effect size, whereas small studies are more likely to be included when they show a relatively large effect size. For the moderation analysis, we performed statistical comparisons between the categories of each moderator (the case of categorical moderators) and meta-regressions for continuous moderators.

The miRNA expression data retrieved from the TCGA and GEO databases were analyzed in GraphPad Prism version 8.2.1(GraphPad Software, San Diego, USA). We first separated the normal tissue from the tumor tissue, and then applied an unpaired two-way T-test for each dataset.

The miRNA targets were discovered by searching the TargetScan online tool, which gave us 384 results. At the same time, we searched the GEPIA2 web server that works with standard processed data from the TCGA and GTEx projects. For the differential expression analysis, the Log_2_FC was set at 1 and the *q*-value cutoff at 0.01. The chosen differential expression method was ANOVA. We then found the common genes for both groups and constructed an interaction network in the String database (https://string-db.org/). The minimum required interaction score was set at low confidence (0.150) and clustered with the MCL clustering with an inflation parameter of 2. The gene functional profiling was done in g:Profiler (https://biit.cs.ut.ee/gprofiler/gost). The Kaplan Meier survival plots for gene expression were calculated for the overall survival Cutoff-High (%) of 75% and Cutoff-Low (%) of 25%, and calculation of hazard ratio based on Cox PH Model. The Pearson correlation between genes and miR-21 was calculated in GraphPad Prism.

## 3. Results

### 3.1. Database Screening and Characteristics of the Included Studies

**Figure 1:**
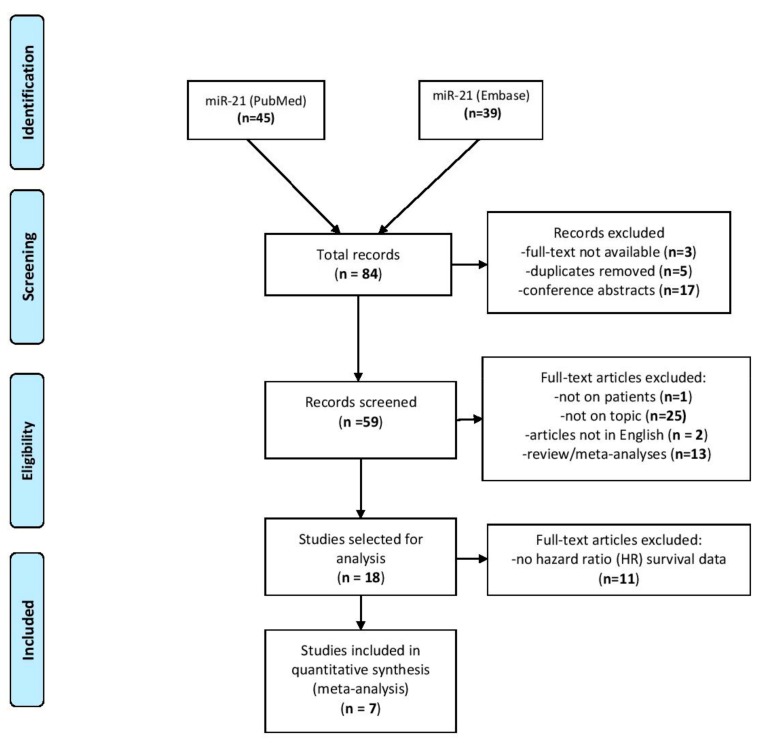
PRISMA flowchart of identification and selection of studies. PRISMA: Preferred Reporting Items for Systematic Reviews and Meta-Analyses.

The identification and selection trial are briefly illustrated in Figure 1. The online search in PubMed and Embase retrieved 84 articles for miR-21 and the HNSCC group of cancers. After the removal of duplicates, we manually excluded conference abstracts and articles with no full-text available. From the remaining 59 articles, we eliminated those that were irrelevant for the analysis, meaning reviews/meta-analysis publications, experiments that were not conducted on patients, and those that were not available in English. Eventually, we removed 11 more articles, as survival analyses was not reported as HR and 95% CI. The current meta-analysis collected seven articles that investigated the prognostic value of miR-21 in head and neck cancers [21,22,23,24,25,26,27].

The basic aspects in the selected studies are represented in Table 1. The present meta-analysis included a total number of 757 patients from six countries (Brazil, Denmark, Korea, Serbia, Taiwan, and USA). The investigations were focused on HNSCC localized in the oropharynx, hypopharynx, larynx, oral cavity, and tongue. All studies used tissue samples to assess the expression of miR-21, and the expression quantification assays included quantitative reverse-transcription PCR (qRT-PCR) and microarray or in situ hybridization (ISH). The involvement of miR-21 in the outcome of HNSCC patients was examined as DFS, OS, RFS, CSS, and 5-year survival.

### 3.2. Data Analysis

**Figure 2:**
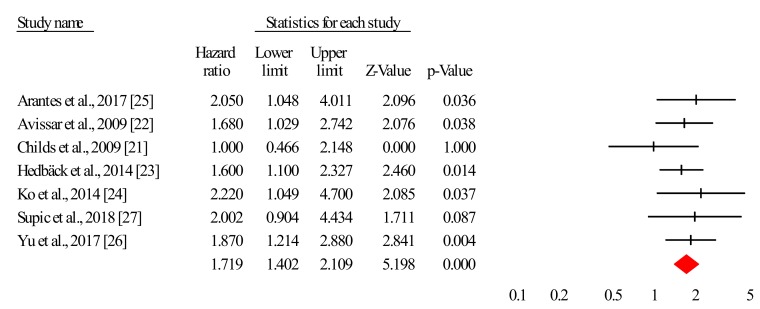
Forest plot for all analyzed studies.

Figure 2 describes the forest plot for miR-21 with the hazard ratio for each study included in the meta-analysis. As shown, from a total of seven studies included in the analysis, five revealed significant hazard ratios [22,23,24,25,26] indicating high expressions of miR-21 associated with poor survival, while two studies obtained nonsignificant hazard ratios [21,27].

The overall corrected hazard ratio is HR = 1.719, 95% CI = (1.402, 2.109) and statistically significant (Z = 5.198, *p* < 0.001), indicating that a high expression of miR-21 is associated with poor survival for patients diagnosed with HNSCC.

The heterogeneity analysis performed for the distribution of effect sizes in our meta-analysis indicated nonsignificant heterogeneity, Q (6) = 3.078, *p* = 0.799. As a consequence, all the data analysis was performed using a fixed-effects model.

### 3.3. Publication Bias Analysis

#### 3.3.1. Classic Fail-safe *N*

In order to test the publication bias, first, we used the Classic Fail-safe N of Rosenthal. The concern of publication bias is that some nonsignificant studies are missing from the analysis, and that these studies, if included, would nullify the observed effect. If this number is relatively small, then there are reasons for concern. However, if this number is large, it generates confidence that the effect size, while possibly inflated by the exclusion of some studies, is nevertheless not null.

Our meta-analysis incorporates data from seven studies, which yields a z-value of 5.015 and a corresponding 2-tailed *p*-value of 0.0001. The fail-safe *N* is 39. This means that we would need to locate and include 39 ‘null’ studies in order for the combined two-tailed *p*-value to exceed 0.05. In other words, there would have to be 5.6 missing studies with a null effect for every observed study for our effect to be nullified.

#### 3.3.2. Begg and Mazumdar’s rank correlation

Our second publication bias analysis was performed using the Begg and Mazumdar’s rank correlation test. This test is concerned with the potential relationship between the size of the studies and the effect size obtained by each one. The results revealed a nonsignificant Kendall’s tau b of 0.095, with a two-tailed *p*-value of 0.763 (based on continuity-corrected normal approximation), suggesting there is no tendency for studies which are more precise (and implicitly larger) to generate larger effect sizes.

### 3.4. Moderators’ Analysis

In our moderation analysis, we tested two categorical moderators (type of sample: FFPE tissue vs tissue, technique: ISH vs. Microarray/qRT-PCR) and five continuous moderators (percentage of women, percentage of smokers, percentage of HPV-16 positive patients, percentage of stage III/IV patients, and percentage of patients who have undergone therapy in the follow-up time, counted as data reported in the studies analyzed).

#### 3.4.1. Categorical moderators

##### Sample Type

As shown in Table 2, the analysis of hazard ratios as a function of sample type revealed significant HR for both types of samples analyzed, HR = 1.771, 95% CI = (1.313, 2.391), for FFPE tissue and HR = 1.675, 95% CI = (1.267, 2.215) for tissue, with no significant differences between those two categories, Q (1) = 0.071, *p* = 0.789.

##### Technique

The analysis performed for this potential moderator revealed significant HR for both types of techniques, HR = 1.767, 95% CI = (1.356, 2.303), *p* < 0.001 for ISH and HR = 1.651, 95% CI = (1.197, 2.277) for Microarray/qRT-PCR, with no significant differences between those two categories, Q (1) = 0.103, *p* = 0.749.

#### 3.4.2. Continuous moderators

In order to test continuous moderators, we performed a metaregression in which the percentage of women, smokers, HPV-16 positive patients, stage III/IV patients, and patients who had undergone treatment after the sampling, were treated as predictors of hazard ratios of miR-21.

##### Percentage of Women

Among the studies analyzed, only six reported the proportion of women. We performed a meta-regression analysis in order to test the predictive value of the proportion of women in the samples upon the hazard ratios. The results revealed that this variable did not have a significant predictive value, B = −0.004, *p* = 0.674.

##### Percentage of Smokers

Among the selected studies, only five reported the proportion of smokers. The analysis of the predictive value of the proportion of smokers in each sample revealed that this variable had a nonsignificant predictive value, B = −0.025, *p* = 0.265.

##### Percentage of HPV-16 Positive Patients

Among the selected studies, only four reported the proportion of HPV-16 positive. The analysis of the predictive value of the proportion of HPV-16 positive patients in each sample revealed that this variable had a nonsignificant predictive value, B = −0.019, *p* = 0.250.

##### Percentage of Patients with Stage III/IV Disease

Only four studies included in the meta-analysis reported the patients separated as either low stage, i.e., I/II stages, or high-stage, i.e., III/IV stages. The analysis of the predictive value of the proportion of stage III/IV patients in each sample revealed that this variable had a nonsignificant predictive value, B = −0.005, *p* = 0.758.

##### Percentage of Patients Who Have Undergone Therapy after the Sampling

Among the selected studies, only three reported the proportion of patients treated after the sampling. The analysis of the predictive value of this variable revealed a nonsignificant predictive value, B = 0.003, *p* = 0.658.

### 3.5. MiR-21 Expression Across Various Datasets

**Figure 3:**
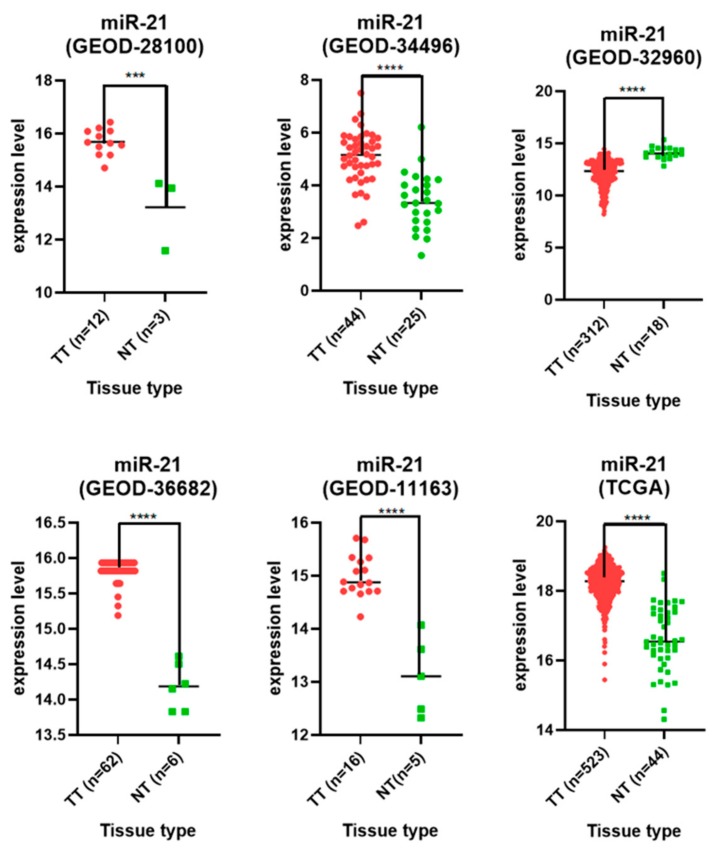
Scatter plot comparison of has-miR-21-5p across 6 datasets.

In our final analysis, we included a total of six independent datasets: five retrieved from GEO, which included microarray analyses done on Agilent or the Affimetrix platform, and one from TCGA, which was done with the help of the miRNASeq technique. The studies included a total number of 969 tumor samples and 101 normal tissue samples. In all cases, the unpaired two-tailed T-test showed *p* < 0.001. In five of the six included databases, miR-21-5p was upregulated in tumor tissue in comparison with normal tissue. In one dataset, however, GEOD-32960, miR-21-5p was down-regulated in tumor tissue. This might be caused by a very small number of normal tissues provided (Figure 3).

#### 3.5.1. miR-21 HNSCC targets are involved in angiogenesis and RUNX expression

**Figure 4:**
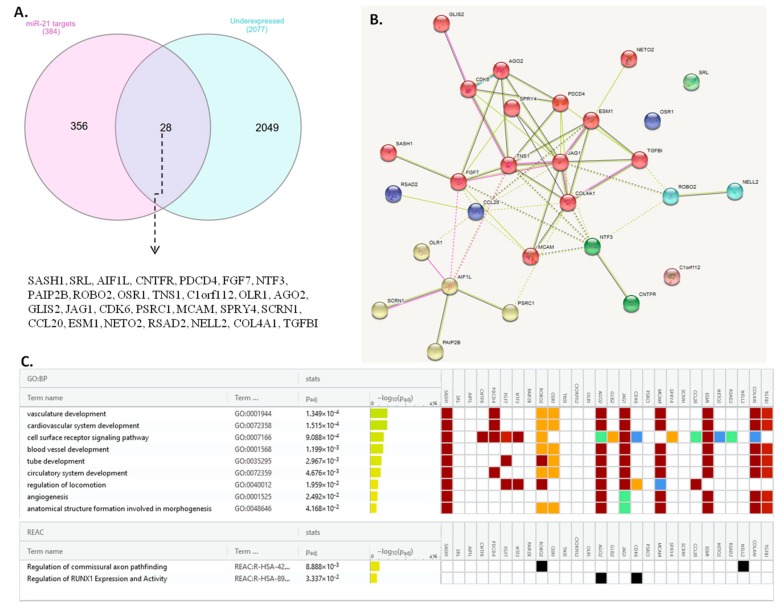
(**A**) Venn diagram of common genes (28) between under-expressed genes in HNSCC tumors and miR-21-5p target genes. (**B**) The STRING protein network of interactions between the 28 common genes; the minimum required interaction score was set at low confidence (0.150) and clustered with the MCL (Markov Cluster Algorithm) clustering with an inflation parameter of 2. (**C**) Gene annotation of the miR-21 potential targets in HNSCC together with associated biological function with the g:Profiler online tool. The genes are involved in vasculature development, cardiovascular system, cell surface receptor signaling pathway, blood vessel development, tube development, circulatory system development, regulation of locomotion, angiogenesis, and anatomical structure formation. Some of these genes also regulate commissural axon pathfinding, and RUNX1 (Runt-related transcription factor 1) expression and activity.

miR-21-5p was discovered to have 384 target genes; a differential gene analysis done in GEPIA2 revealed that there are 2077 under-expressed genes in HNSCC tumor tissue versus normal tissue. From these two lists of genes, 28 genes were found to be common (Figure 4A). The list of common genes is presented in Table 3.

Protein–Protein Interaction Networks Functional Enrichment Analysis (STRING) shows that the panel of 28 genes have a weak level of interaction with each other (Figure 4B), forming functional clusters only at when the interaction score is set at minimum levels of 0.150 (low confidence). According to the Gene ontology database included in the g:Profiler online tool, the 28 genes that are both targets of miR-21-5p and are under-expressed in HNSCC tissue are associated with the following biological functions: vasculature development (10 genes: *SASHI1, PDCD4, ROBO2, OSR1, AGO2, JAG1, MCAM1, ESM1, COL4A1*, and *TGFΒI*), cardiovascular system development (10 genes—same as in the case of vasculature development), cell surface receptor signaling pathway (16 genes: *SASH1, CNTFR, PDCD4, FGF7, NTF3, ROBO2, AGO2, GLIS2, JAG1, CDK6, SPRY4, CCL20, ESM1, NETO2, RSAD2*, and *COL4A1*), blood vessel development (9 genes*: SASHI1, ROBO2, OSR1, AGO2, JAG1, MCAM1, ESM1, COL4A1*, and *TGFΒI*), tube development (10 genes: *SASH1, FGF7, ROBO2, OSR1, AGO2, JAG1, MCAM, ESM1, COL4A1*, and *TGF ΒI*), circulatory system development (10 genes: *SASH1, PDCD4, ROBO2, OSR1, AGO2, JAG1, MCAM, ESM1, COL4A1*, and *TGF ΒI*), regulation of locomotion (9 genes: *SASH1, FGF7, NTF3, ROBO2, AGO2, JAG1, CDK6, MCAM*, and *CCL20*), angiogenesis (*SASH1, AGO2, JAG1, MCAM, ESM1, COL4A1*, and *TGF ΒI*) and anatomical structure formation involved in morphogenesis (9 genes: *SASH1, ROBO2, OSR1, AGO2, JAG1, MCAM, ESM1, COL4A1*, and *TGF ΒI*). According to the Reactome database, *ROBO2* and *NELL2* are involved in the regulation of commissural axon pathfinding, and *AGO2* and *CDK6* are involved in RUNX1 expression and activity. As follows, the genes most probably targeted by miR-21-5p in HNSCC are involved mainly in the function of the blood vessel development and regulation. According to −log_10_(padj) values, their involvement in the above processes are weakly supported statically (Figure 4C).

#### 3.5.2. Targeted gene expression correlation with overall survival

**Figure 5:**
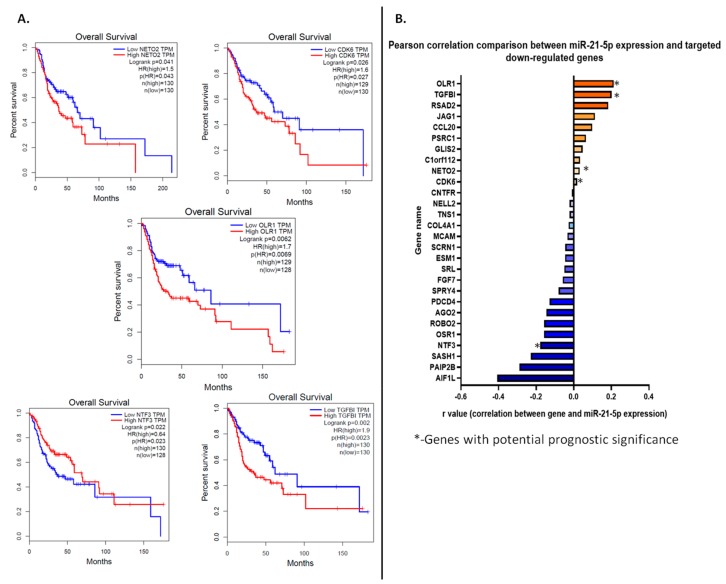
(**A**) Kaplan-Meier survival plots for miR-21 targets with statistically significant correlation with overall survival rate of HNSCC patients. These are: *NETO2*, *CDK6*, *OLR1*, *NTF3*, and *TGFBI*. (**B**) “r” values resulted from Pearson correlation analysis between miR-21 expression value and the expression of 28 potential targeted genes in HNSCC.

From the 28 common genes, only 5 showed a statistically-significant correlation with the overall survival rate of HNSCC patients (Log rank *p* < 0.05). These genes are *NETO2*, *CDK6*, *OLR1*, and *NTF3*, *TGFBI* (Figure 5A). HNSCC patients with low expression of *NETO2, CDK6, OLR1*, and *TGFBI* have a better overall survival rate than HNSCC patients with high expression of these genes, while patients with low expression of NTF3 have a worse overall survival rate than those with high expression of NTF3. This means that even though TCGA data shows that *NETO2, CDK6, OLR1*, and *TGFBI* are underexpressed in HNSCC tissue compared to normal adjacent one, patients who show a low expression of this gene have a better disease outcome with regard to overall survival rate. Therefore, the data highlights a more complex signaling mechanism, which is not limited only to the interaction between miR-21 and the predicted target genes in HNSCC. The Pearson correlation analysis between the expression of all 28 genes from Table 3 and miR-21 expression shows that within the five genes with an impact on the overall survival of HNSCC patients, four have positive correlations with miR-21 (ORL1, TGFBI, NETO2, and CDK6) (see Figure 5B). The data might suggest that these are not true miR-21-5p targets in HNSC, because the expression of these genes has a dependent increase with the increase of miR-21-5p expression. The *AIF1L* gene showed the most negative r value of correlation with miR-21; however, this gene does not have a significant impact upon the overall survival of HNSCC patients. Only one gene passed all the evaluations: *NTF3*. This gene is underexpressed in HNSCC tumor tissue versus normal adjacent tissue, it is targeted by miR-21-5p, according to the TargetScan online tool, its low expression in tumor tissue reduces the overall survival rate of HNSCC patients, and it has a negative correlation with miR-21 expression.

## 4. Discussion

Accumulating data suggest that aberrantly-expressed miR-21 is a mark of malignant phenotype in several cancers [28], including HNSCC [29], where, even if the pathologies forming this group are very different in terms of histology or anatomic site, this microRNA is upregulated in the vast majority of cases. There are no disputes regarding the importance of miR-21 as a cancer-related transcript. Furthermore, there are studies showing that a combination of miR-21 and miR-375 expression profiles has a great potential to discriminate patients with oral tongue squamous cell carcinoma from normal controls [30]. The present meta-analysis brings direct confirmation of the oncogenic role of miR-21 in head and neck cancers. Using fixed-effects model, the investigation showed that miR-21 upregulation predicts shorter survival in HNSCC patients. We also found that the expression of miR-21 in the TCGA and GEO datasets is upregulated in the majority of cases, and that the underexpression of some of its targets is associated with worse outcome in terms of survival rates. Our results confirm the oncogenic potential of miR-21 in head and neck cancers, given the fact that in this category of cancers, the anatomic sites include many different histological types, and the upregulation of miR-21 remains significant and allows the separation of these patients’ outcomes. A comparison between reported continuous variables that could influence the predictive value of miR-21 in these patients was not possible, as the selected studies did not report separate HRs for each group, i.e., males vs. females, smokers vs. nonsmokers, stage I/II vs. III/IV, HPV-16+ vs. HPV-16-, and treated vs. untreated patients; therefore, we investigated the moderator effect—evaluated as proportions in the cohort—of these variables on the predictive value of miR-21 in HNSCC patients. When continuous moderators were assessed, our analysis revealed that the prognostic value of miR-21 expression remains stable, that it is not moderated by the percentage of women, smokers, stage III/IV patients, and HPV-16 status, and that patients who underwent conventional chemo- or/and radiotherapy in the sample did not influence the predictive value of miR-21. Given that the studies reported the proportion of patients that followed a treatment course after the moment of the surgical resection of the tissue sample, these results should be interpreted with caution. Therefore, all these investigations confirm the reliable nature of miR-21 in assessing the outcome of HNSCC patients.

**Figure 6:**
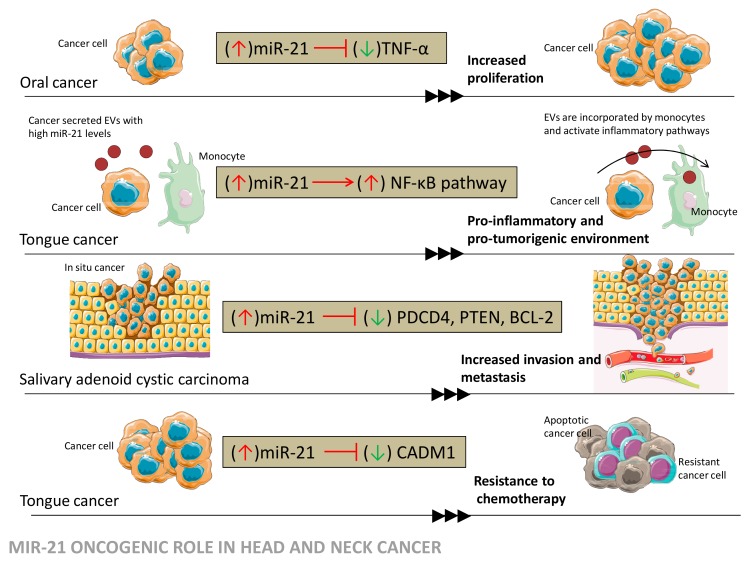
MiR-21 oncogenic role in head and neck cancers according to the data presented in the literature. miR-21 supports cancer development through the inhibition of tumor suppressor genes and the activation of the oncogenic signaling involved in proliferation, pro-inflammatory environments, invasion and metastasis, and resistance to chemotherapy.

The upregulation of miR-21 was evaluated in HNSCC lines, revealing its involvement in cellular growth and cell cycle regulation, and its function as a regulatory molecule being exerted by complementary binding to the 3’ UTR of PDCD4, with similar observations in HPV+ and HPV- cell lines [31]. It has been shown that in oral cancer, miR-21 is involved in the regulation of proliferation in cancer cells by inhibition of tumor necrosis factor alpha (*TNF-α*), and in the upregulation of miR-21, resulting in increased proliferation and decreased *TNF-α* expression [32]. The oncogenic potential of miR-21 in oral squamous cell carcinoma (OSCC) cell lines was underlined in relation with PTEN protein levels. Specifically, the induced underexpression of miR-21 correlated with the upregulation of *PTEN* gene expression and inhibition of cell growth, with these results showing miR-21 to be a potential therapeutic target in OSCC [33]. MiR-21 was also reported as a regulator of the immune response in tongue cancer, playing a role in expressly activating the nuclear factor NF-κB pathway in CAL27 cell line exosomes [34]. In salivary adenoid cystic carcinoma, by targeting genes such as *PDCD4*, *PTEN*, and *BCL*-2, miR-21 regulates the invasion and metastasis potential of the SACC-LM cell line [16]. In nasopharyngeal carcinoma, in vitro studies pointed out that the mechanism by which miR-21 controls the apoptosis, invasion, and cellular growth involves the targeting of the *PTEN* gene, thus interfering with the Akt pathway and *BCL-2* expression [35,36]. The studies also revealed details related to the mechanism of miR-21 regulation. Precisely, the promoter of miR-21 requires the direct binding of STAT3 protein in order to initiate the transcription of this molecule [35]. Resistance to therapy is also mediated by miR-21, as, in tongue cancer, it was reported that in the Tca8113 and SCC-25 cell lines, transfection with miR-21 mimic resulted in enhanced chemoresistance capacity coupled with reduced apoptosis. MiR-21 interferes with resistance to chemotherapy by binding to cell adhesion molecule-1 (*CADM1*) mRNA [37]. Figure 6 summarizes the mechanisms behind the oncogenic role of miR-21 in malignancies from the head and neck area, according to the data presented in the scientific literature. Our bioinformatics analysis supports the oncogenic nature of miR-21 via the targeting of *NTF3* and the association with a decreased survival rate in HNSCC patients.

The association between human papillomaviruses (HPV), and particularly HPV-16 and NCSCC, is widely acknowledged, its carcinogenicity being proven previously [38,39], but also recognized by the International Agency for Research on Cancer (IARC) in 2007 as a risk factor for the development of oropharyngeal carcinoma [40]. There are reports indicating that the prognostic role of miR-21 is stronger in HNSCC patients with HPV-negative status tumors. Keeping in mind that miR-21 targets molecules that interfere with p53 pathways, this could be explained by the fact that in HPV-positive tumors, the p53 protein expression is not altered, being more sensitive to chemo- or/and radiotherapy [24,41]. Our study revealed that HPV status did not moderate the prognostic value of the expression levels of miR-21, indicating that this molecule may act as a stable prognostic marker for the survival of patients diagnosed with HNSCC. Therefore, more details regarding the characteristics of these patients should be provided in each study to facilitate data accessibility in order to obtain reliable results in this respect. 

## 5. Conclusions

In conclusion, using fixed effects model, our meta-analysis was successfully underlined the potential of miR-21 and its stability as prognostic biomarker in head and neck squamous cell carcinoma (HNSCC) patients. Our results confirm the oncogenic nature of this transcript in this group of malignancies. Taking into consideration the diversity of these cancers, the oncogenic effect of miR-21 is undeniable, with a bioinformatics analysis confirming the interaction with genes with known involvement in carcinogenesis. The current meta-analysis was successful at confirming the prognostic biomarker potential of miR-21 in HNSCC patients, with highlights regarding the oncogenic role of this transcript observed in HNSCC cell lines. However, in order to transfer its utility in clinical trials with the ultimate purpose of developing personalized treatments for these patients, standardization of the used methodologies and of the clinical data that should be published is required, together with deciphering the molecular processes that lead to the abnormal expression of miR-21 and its downstream consequences in this group of cancers.

## Figures and Tables

**Table 1:** Baseline characteristics of the selected studies evaluating the prognostic value of miR-21 in HNSCC patients.

Reference	Country	Anatomic Site (*n*)	*n*	Smoking Status	HPV Status (*n*)	Outcome	Effect Sizes
Arantes et al. 2017 [25]	Brazil	Oropharynx (35) Hypopharynx/larynx (36)	71	yes: 57 no: 14	Positive (17)	OS	HR: 2.05 (1.05–4.02)
Ko et al. 2014 [24]	Korea	Oral cavity (88) Oropharyngeal (79)	167	yes: 109 no: 57	Positive (36)	RFS	HR: 1.659 (0.824–3.343)
						CSS	HR: 2.972 (1.34–6.59)
Hedbäck et al. 2014 [23]	Denmark	Oral cavity (86)	86	-	-	DFS	HR: 1.6 (1.1–2.5)
Avissar et al. 2009 [22]	USA	Oral cavity (94) Pharyngeal (31) Laryngeal (22)	169	yes: 120 no: 22	Positive (19)	5-year survival	HR: 1.68 (1.04–2.77)
Childs et al. 2009 [21]	USA	Oral cavity (31) Oropharynx (32) Hypopharynx (9) Larynx (32)	104	yes: 46 no: 56	Positive (37)	OS	HR: 1
Yu et al. 2017 [26]	Taiwan	Oral cavity (100)	100	-	-	DFS	HR: 1.87 (1.21–2.87)
Supic et al. 2018 [27]	Serbia	Tongue (60)	60	yes: 42 no: 18	-	OS	HR: 2.002 (0.904–4.434)

OS: overall survival; RFS: relapse-free survival; CSS: cancer-specific survival; DFS: disease-free survival; HNSCC: head and neck squamous cell carcinoma.

**Table 2:** The analysis performed for categorical moderators.

Moderator	Categories of the Moderator	No. of Studies	Hazard Ratio	Lower Limit	Upper Limit	QB	df	*p*
Sample	FFPE tissue	3	1.771	1.313	2.391	0.071	1	0.789
	Tissue	4	1.675	1.267	2.215			
Technique	ISH	3	1.767	1.356	2.303	0.103	1	0.749
	Microarray/qRT-PCR	4	1.651	1.197	2.277			

FFPE: formalin-fixed paraffin embedded; QB: between-groups heterogeneity; df: degrees of freedom.

**Table 3:** The list of common genes between miR-21-5p targets and genes under-expressed in HNSCC tumors versus normal adjacent tissue).

Full Name	Abbreviation
Argonaute RISC Catalytic Component 2	AGO2
Allograft Inflammatory Factor 1 Like	AIF1L
Chromosome 1 Open Reading Frame 112	C1orf112
C-C Motif Chemokine Ligand 20	CCL20
Cyclin Dependent Kinase 6	CDK6
Ciliary Neurotrophic Factor Receptor	CNTFR
Collagen Type IV Alpha 1 Chain	COL4A1
Endothelial Cell Specific Molecule 1	ESM1
Fibroblast Growth Factor 7	FGF7
GLIS Family Zinc Finger 2	GLIS2
Jagged Canonical Notch Ligand 1	JAG1
Melanoma Cell Adhesion Molecule	MCAM
Neural EGFL Like 2	NELL2
Neuropilin And Tolloid Like 2	NETO2
Neurotrophin 3	NTF3
Oxidized Low Density Lipoprotein Receptor 1	OLR1
Odd-Skipped Related Transcription Factor 1	OSR1
Poly(A) Binding Protein Interacting Protein 2B	PAIP2B
Programmed Cell Death 4	PDCD4
Proline And Serine Rich Coiled-Coil 1	PSRC1
Roundabout Guidance Receptor 2	ROBO2
Radical S-Adenosyl Methionine Domain Containing 2	RSAD2
SAM And SH3 Domain Containing 1	SASH1
Secernin 1	SCRN1
Sprouty RTK Signaling Antagonist 4	SPRY4
Sarcalumenin	SRL
Transforming Growth Factor Beta Induced	TGFΒI
Tensin 1	TNS1
